# Integrating Computational and Experimental Approaches for the Discovery of Multifunctional Peptides from the Marine Gastropod *Pisania pusio* with Antimicrobial and Anticancer Properties

**DOI:** 10.3390/md24010032

**Published:** 2026-01-08

**Authors:** Ernesto M. Martell-Huguet, Thalia Moran-Avila, José E. Villuendas, Armando Rodriguez, Ann-Kathrin Kissmann, Ludger Ständker, Sebastian Wiese, Anselmo J. Otero-Gonzalez, Frank Rosenau

**Affiliations:** 1 Institute of Pharmaceutical Biotechnology, Ulm University, 89081 Ulm, Germany; nestmartell@gmail.com (E.M.M.-H.); ann-kathrin.kissmann@uni-ulm.de (A.-K.K.); 2 Center for Protein Studies, Faculty of Biology, University of Havana, 25 and I, La Habana 10400, Cuba; moravilat@gmail.com (T.M.-A.); joseenriquevilluendasquesada@gmail.com (J.E.V.); 3 ULMTec Core Facility of Mass Spectrometry and Proteomics, Faculty of Medicine, Ulm University, Albert-Einstein-Allee 11, 89081 Ulm, Germany; armando.rodriguez-alfonso@uni-ulm.de (A.R.); sebastian.wiese@uni-ulm.de (S.W.); 4 ULMTec Core Facility for Functional Peptidomics, Ulm Peptide Pharmaceuticals (U-PEP), Faculty of Medicine, Ulm University, 89081 Ulm, Germany; ludger.staendker@uni-ulm.de

**Keywords:** marine mollusks, antimicrobial peptides, anticancer activity, in silico predictions

## Abstract

Marine invertebrates are a prime source of biologically active peptides due to their role in humoral immunity. These peptides typically exhibit broad-spectrum functions, including antibacterial, antifungal, anticancer, and immunomodulatory activities. In this report, we describe the identification and biological characterization of five novel bioactive peptides from the marine mollusk *Pisania pusio*. An extract of *P. pusio* was analyzed using nanoLC-ESI-MS-MS, and five peptides (PP1–5) were selected via bioinformatic screening as potential antimicrobial and anticancer peptides and subsequently validated experimentally. Among these, PP1, PP2, and PP4 were identified as cryptides derived from the proteolytic cleavage of actin, while PP3 and PP5 are novel peptides with no known protein precursors. All peptides exhibited moderate activity against *Pseudomonas aeruginosa*, *Escherichia coli*, *Staphylococcus aureus*, and *Klebsiella pneumoniae* with minimum inhibitory concentrations (MICs) predominantly at 100 µM. In contrast, only PP1 and PP5 were active against cancer cells, with PP1 being the most effective against A375 melanoma cells (IC_50_ = 17.08 µM). This experimental validation confirmed the utility of the integrated in silico/peptidomic pipeline for lead identification. None of these peptides showed significant hemolytic activity or toxicity on fetal lung fibroblasts over 800 μM, demonstrating promising in vitro selectivity. These results highlight the multifunctional nature of *P. pusio*-derived peptides and their potential as lead compounds for further optimization and development into therapeutic agents against microbial infections and cancer, subject to more comprehensive safety evaluations in relevant models

## 1. Introduction

The convergence of two major global health crises, the escalating pandemic of antimicrobial resistance (AMR) and the limitations of conventional cancer therapies, has created an urgent need for novel therapeutic agents with dual activity and new mechanisms of action that can circumvent existing resistance pathways [1,2,3,4,5,6,7]. This is particularly critical for immunocompromised patients, such as those undergoing chemotherapy, who face heightened infection risks amid diminishing treatment options [5,6]

In response to this dual challenge, Host Defense Peptides (HDPs), also known as Antimicrobial Peptides (AMPs), have emerged as a promising class of biomolecules [8]. These small, gene-encoded peptides (typically 12–50 amino acids) are key effectors of the innate immune system [9]. Their net positive charge and amphipathic structure allow them to interact with and disrupt the negatively charged membranes of pathogens and cancer cells, a mechanism that reduces the likelihood of resistance development compared to conventional drugs [10,11]. Beyond their direct membranolytic activity, many HDPs exhibit immunomodulatory functions, such as modulating cytokine responses and promoting wound healing, which adds a valuable layer to their therapeutic profile [12]. Crucially, a subset of these peptides, termed Anticancer Peptides (ACPs), can selectively target malignant cells based on their distinct membrane composition, offering a template for developing safer, more effective oncology treatments [13,14].

Marine invertebrates represent a premier source for discovering novel bioactive peptides [15]. As organisms lacking adaptive immunity, they rely on a sophisticated innate immune system, producing a diverse arsenal of peptides as a first line of defense [16,17]. The marine environment itself acts as a unique crucible for bioactivity; constant exposure to a high-density microbial load in a hypertonic environment has driven the evolution of peptides that are potent, stable, and often salt-resistant, a significant advantage over many terrestrial counterparts whose activity is inhibited by physiological salt concentrations [18,19]. While peptides from mollusks like mussels (*Mytilidae*) and cone snails (*Conidae*) have been relatively well-studied, the vast majority of molluscan diversity remains unexplored [20,21]. Several species from the *Muricidae* and *Buccinidae* families are known for their rich venoms and defensive secretions; yet, only a small fraction of the entire *Neogatropoda* order has been studied for the isolation of bioactive compounds, and their peptide repertoire is largely uncharacterized [22]. This gap highlights a significant opportunity for biodiscovery, as each unexplored species may produce unique peptide scaffolds with optimized functions against specific ecological pressures [23]. Beyond genetically encoded HDPs, bioactive peptides can also originate from the proteolytic processing of ubiquitous structural or functional proteins, yielding cryptic bioactive fragments known as cryptides. These peptides, though not products of dedicated defense genes, can exhibit potent antimicrobial and anticancer activities, expanding the repertoire of defensive molecules in marine organisms [24,25].

**Figure 1:**
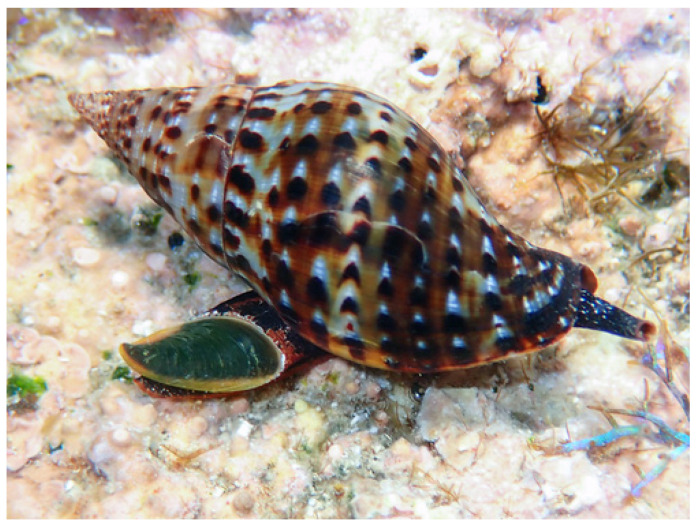
*Pisania pusio* (Linnaeus, 1758). Occurrence data accessed through GBIF (GBIF Ocurrence Download https://www.inaturalist.org/observations/197986153, accessed on 20 May 2025). Original, unmodified photograph taken in Saint Kitts and Nevis by pkondrashov (https://www.inaturalist.org/photos/348849010, accessed on 20 May 2025, licensed under http://creativecommons.org/licenses/by-nc/4.0/). The species used in this study is not endangered and is widely distributed in the region.

Within this context, the marine gastropod *Pisania pusio* (Linnaeus, 1758, Order: *Neogastropoda*, family *Pisaniidae*, Figure 1) emerges as a compelling candidate for peptide discovery. Despite the ecological success and wide distribution of the gastropods of the family *Pisaniidae*, their peptidome has never been systematically investigated. This gap is particularly striking given the demonstrated biomedical potential of peptides from related gastropod families [26,27]. It was thus investigated whether the distinct pathogenic and environmental pressures on *P. pusio* drive the production of a unique suite of bioactive peptides with antimicrobial and anticancer activities. To address this, an integrated discovery pipeline was employed, combining advanced peptidomics and in silico prediction. This work reports the identification and comprehensive characterization of five novel peptides (PP-1 to PP-5) from *P. pusio*. The peptides were rigorously evaluated for their antimicrobial potency against a panel of clinically relevant bacteria, their cytotoxic activity against human cancer cell lines, and their safety profile through hemolytic and toxicity assays. This study not only validates *P. pusio* as a valuable source of multifunctional peptides but also identifies specific lead compounds, particularly PP-1 and PP-5, with significant potential for development into novel therapeutic agents against infections and cancer.

## 2. Results

### 2.1. In Silico Profiling Reveals Non-Toxic Pisania pusio Peptides with Dual Antimicrobial and Anticancer Potential

The sequences of the five *P. pusio*-derived peptides were submitted to in silico analysis to predict their physicochemical properties (Table 1) and possible antimicrobial and anticancer effects (Table 2). According to bioinformatics analyses by ClassAMP [28], CAMPR3 [29], and AMP Scanner vr.2 [30] tools, PP1, PP3, and PP5 have a high antibacterial potential. Similarly, analyses by AntiCP 2.0 [31] and CancerPPD2 [32] servers revealed the potential of PP1, PP2, PP3, and PP5 as anticancer peptides, highlighting PP1 as the peptide with the highest score in both tools.

In silico predictions with ToxinPred [33] and CellPPD [34] suggested the safety profile of the peptides, as no toxicity or cell-penetrating activity was predicted for any of the five sequences. Furthermore, in silico prediction of proteolytic stability in an intestine-like environment using the HLP server [35] indicated that all peptides exhibited predicted high stability under proteolytic conditions, particularly PP-3 and PP-5, which had a predicted half-life exceeding two seconds. The five peptides (PP1–5) were synthesized with C-terminal amidation to enhance their stability against carboxypeptidases.

Furthermore, BLAST (version 2.17.0) analysis with Uniprot and NCBI web interfaces against the corresponding *Mollusca* protein databases revealed that peptides PP1, PP2, and PP4 show 100% sequence identity to conserved regions of actin proteins from various gastropods and bivalves (see Supplementary Figure S1). This strongly suggests these peptides are cryptic bioactive fragments (cryptides) generated from the proteolytic cleavage of this abundant structural protein, rather than products of dedicated host-defense genes

The calculated biophysical parameters for the PP peptides are summarized in Supplementary Table S1. The peptides exhibited variable interfacial hydrophobicity (ΔG-Interface = +0.64 to +5.67 kcal/mol) and hydrophobic moment values (µH = 0.29 to 0.48). The protein-binding potential (Boman index) ranged from −0.52 to 2.53 kcal/mol. Notably, the active peptides PP1 and PP5 displayed the most favorable (lowest positive) ΔG-Interface values among the set, alongside negative Boman indices.

### 2.2. P. pusio-Derived Peptides Exhibit Low Hemolytic Activity

**Figure 2:**
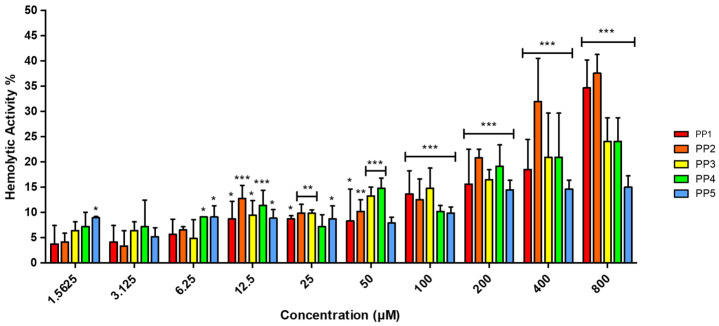
Percentage of hemolytic activity of PP peptides in hRBC determined by hemoglobin release. A range of peptide concentrations (1.5625–800 μM) was evaluated against hRBC for 2 h. Phosphate-buffered saline (PBS) and Triton X-100 at 1% were used as negative (0% hemolysis) and positive controls (100% hemolysis), respectively. The results are representative of three independent experiments, analyzed via one-way ANOVA with α = 0.05 to assess the differences between the peptide concentrations and the negative control (* *p* < 0.05, ** *p* < 0.01, and *** *p* < 0.001).

Hemolytic activity represents a well-documented therapeutic challenge for ACPs, especially for highly hydrophobic variants operating through membranolytic mechanisms [36]. To assess this potential liability, the hemolytic activity of the *P. pusio*-derived peptides was evaluated by hemoglobin release assay. The results demonstrated low selectivity of the peptides toward human red blood cells (hRBC), with hemolytic activity not exceeding 20% for any peptide across the entire concentration range tested (Figure 2).

### 2.3. P. pusio-Derived Peptides Show Moderate Antimicrobial Activity Against Pathogenic Bacteria at High Concentrations

The experimental determination of the minimum inhibitory concentration (MIC) against a panel of clinically relevant bacteria reveals moderate activity profiles among the *P. pusio*-derived peptides (Table 3). PP-1 stands out as the most potent and broad-spectrum peptide, exhibiting complete growth inhibition against all four bacterial strains tested. Notably, it showed the highest potency against the Gram-positive *Staphylococcus aureus* (MIC = 25 μM), while maintaining consistent activity against the Gram-negative pathogens *Pseudomonas aeruginosa*, *Klebsiella pneumoniae*, and *Escherichia coli* (MIC = 100 μM). PP-5 displayed intermediate activity, being effective against the three Gram-negative strains (MIC = 100 μM) but showing no antimicrobial effect against *S. aureus* at the maximum concentration tested (>100 μM). 1, peptides PP-2, PP-3, and PP-4 demonstrated a limited spectrum of action, with activity observed only against *P. aeruginosa* and *E. coli* (MIC = 100 μM), and no detectable antimicrobial effect against *K. pneumoniae* or *S. aureus* (>100 μM).

### 2.4. Peptides PP-1 and PP-5 Exhibit Selective Cytotoxicity Against Cancer Cell Lines

**Figure 3:**
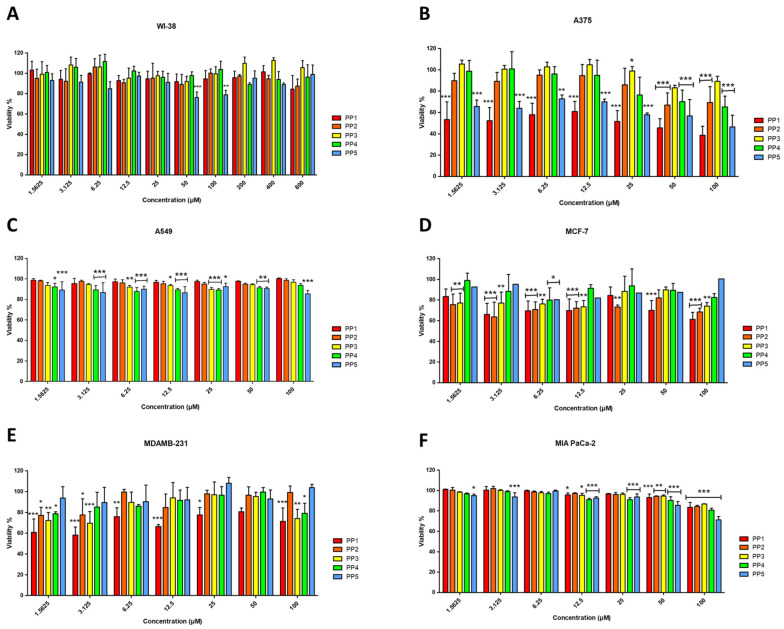
Effects of PP peptides on cell viability of fetal lung fibroblasts WI-38 (**A**), malignant melanoma A375 (**B**), epithelial cell lung carcinoma A549 (**C**), luminal A breast adenocarcinoma MCF-7 (**D**), triple negative basal breast adenocarcinoma MDA-MB-231 (**E**), and pancreatic carcinoma MIA PaCa-2 (**F**), determined by resazurin reduction assay at 24 h of incubation. Cells treated with culture medium served as the negative control (100% viability), while cells treated with 1% Triton X-100 served as the positive control. The results shown are representative of three independent experiments and were analyzed using one-way ANOVA with α = 0.05 to assess differences between peptide concentrations and the negative control for each cell line (* *p* < 0.05, ** *p* < 0.01, and *** *p* < 0.001).

The cytotoxicity profile of the *P. pusio*-derived peptides was evaluated against a panel of five human cancer cell lines and the non-malignant fetal lung fibroblast cell line WI-38 (Figure 3). Dose-response curves obtained from resazurin assays at 24 h quantitatively confirm that peptides PP-1 and PP-5 are the only ones that exert a potent and selective cytotoxic effect, primarily against the A375 malignant melanoma cell line (Figure 3B).

PP-1 emerges as the most potent peptide, showing significant cytotoxicity against the A375 melanoma cell line with an IC_50_ of 17.08 ± 1.36 μM (Table 4). This activity is characterized by a clear concentration-dependent decrease in cell viability. PP-1 also induced a significant, though less potent, reduction in viability in the triple-negative breast adenocarcinoma MDA-MB-231 and the luminal A breast cancer MCF-7 cell lines (Figure 3D,E), with IC_50_ values of 76.25 μM and 98.53 μM, respectively.

PP-5 exhibited a highly selective activity profile, showing significant cytotoxicity exclusively against A375 melanoma cells (IC_50_ = 39.97 ± 1.29 μM) in a dose-dependent manner (Figure 3B), while proving inactive against all other cancer lines and normal cells. Critically, the anticancer activity of PP-1 and PP-5 is coupled with a preliminary indication of selectivity, as none of the peptides significantly reduced the viability of non-malignant WI-38 fibroblasts, even at the highest concentration tested (Figure 3A). This absence of toxicity in this specific normal cell line at concentrations active against cancer cells, combined with the low hemolytic activity, constitutes an initial safety profile that requires further validation in a broader panel of primary cells and tissues. Conversely, peptides PP-2, PP-3, and PP-4 displayed no relevant cytotoxic effects on any malignant cell line (IC_50_ > 100 μM; Figure 3B–F).

### 2.5. Quantitative Profiling of in Vitro Selectivity

To provide a rigorous, quantitative assessment of the therapeutic potential of the *P. pusio*-derived peptides, we performed extended concentration-range assays for the key toxicity endpoints. Neither hemolysis nor cytotoxicity in non-malignant WI-38 fibroblasts reached 50% at the maximum tested concentration of 800 µM (Figure 2 and Figure 3A). Consequently, the standard half-maximal toxicity values (HC_50_ for hemolysis and IC_50_ for WI-38 cells) are >800 µM for all peptides. Given this, traditional selectivity indices based on exact HC_50_ or IC_50_ ratios could not be calculated. Instead, we determined conservative minimum selectivity indices using the 800 µM threshold as a robust lower-limit proxy for significant toxicity. This approach provides a stringent, worst-case estimate of the in vitro therapeutic window.

For antimicrobial activity, the Minimum Hemolytic Selectivity Index (HSI-min) was calculated as: HSI-min = 800 µM/MIC. This index represents the minimum fold-difference between the concentration causing significant hemolysis (HC_50_ > 800 µM for all peptides) and the antimicrobial effective concentration.

For the anticancer lead peptides PP1 and PP5, the Minimum Cytotoxic Selectivity Index (CSI-min) was calculated: CSI-min = 800 µM/IC_50_ (A375). The CSI-min represents the minimum window between toxicity to normal fibroblasts and efficacy against melanoma cells. The results of this analysis are presented in Table 5 and Table 6.

## 3. Discussion

The escalating global health crises of antimicrobial resistance and the inherent limitations of conventional cancer chemotherapies necessitate the exploration of novel therapeutic agents with distinct mechanisms of action [1,7]. Marine invertebrates, which rely on an evolutionarily refined humoral immune system for defense, constitute a prolific source of bioactive peptides characterized by their structural diversity and multifunctionality [15,16,17,18]. This study reports the discovery and functional characterization of five novel peptides derived from the marine gastropod *Pisania pusio*, a species from the hitherto unexplored *Pisaniidae* family. Our approach combined an untargeted peptidomic analysis of the mollusk’s extract via nanoLC-ESI-MS/MS with a subsequent in silico bioinformatic screening of the identified sequences. This integrated discovery pipeline allowed us to efficiently filter a complex peptidomic dataset and select specific candidates for synthesis and testing based on predicted bioactivity, thereby focusing experimental resources on the most promising leads. This strategy identified two peptides, PP1 and PP5, which exhibit antimicrobial and anticancer activities alongside a favorable selectivity profile. It is important to note that the antimicrobial potency of the current peptides, with MICs at 100 µM for most targets, is moderate compared to some clinical-stage antimicrobial peptides. This highlights potency, rather than selectivity, as the primary parameter for improvement in future optimization campaigns aimed at enhancing their therapeutic potential.

The in silico profiling served as a critical step to rationalize the selection of candidates from the numerous sequences obtained through peptidomics. While the computational predictions were valuable for the initial triage and prioritization of peptides for synthesis from a larger dataset, a direct comparison reveals both alignments and notable discrepancies with the experimental outcomes. For instance, the antibacterial predictions for PP1 and PP5 by tools like CAMPR3 were consistent with their experimental activity against Gram-negative pathogens, and the high scores for PP1 as an anticancer peptide (ACP) aligned with its cytotoxicity against A375 melanoma cells (IC_50_ = 17.08 µM) [29,30,31,32]. Furthermore, the predictions of low toxicity and non-cell-penetrating potential correctly foreshadowed the experimental safety data, which showed minimal hemolysis (HC_50_ > 800 µM) and no significant cytotoxicity in non-malignant WI-38 fibroblasts at bioactive concentrations (Figure 2 and Figure 3A) [33,34].

However, important inconsistencies were also observed, underscoring the current limitations of predictive tools. PP1 was classified as “No AMP” by the AMP Scanner vr.2 server [30] despite showing the most relevant experimental MIC (25 µM against S. aureus). Conversely, PP3 and PP5 were predicted as AMPs by multiple tools but exhibited only moderate activity (MIC ≥ 100 µM) against the tested bacterial panel. These discrepancies highlight that while in silico tools are highly useful for identifying potential bioactive sequences and filtering out likely inactive ones in a discovery workflow [37,38], they cannot yet reliably predict the exact antimicrobial spectrum or quantitative potency (MIC values) of novel peptides [39]. Therefore, these platforms are best viewed as effective for de-risking early-stage candidate selection, with experimental validation remaining indispensable for defining true bioactivity and potency.

The differential bioactivity observed among the peptides can be interpreted through the lens of their physicochemical characteristics (Table 1) [40,41]. PP1 is characterized by a neutral net charge, high hydrophobicity (GRAVY = 0.59), and pronounced amphipathicity (0.62). This profile is consistent with established models for membranolytic peptides [10,13]. Its neutral charge is atypical for classical cationic AMPs but is frequently observed in ACPs that target the elevated anionic phosphatidylserine content and increased membrane fluidity of cancer cells [13,36]. The high hydrophobicity and amphipathicity of PP1 are critical for partitioning into and destabilizing lipid bilayers, suggesting a mechanism that may involve the carpet model, in which peptides accumulate parallel to the membrane surface, disrupting lipid packing and causing transient micellization in a detergent-like manner [10]. The sharp, concentration-dependent response curve of PP1 against A375 cells is indicative of such a threshold-dependent membranolytic mechanism.

In contrast, PP5 possesses a distinct profile with a positive charge, moderate hydrophobicity (GRAVY = 0.50), and lower amphipathicity (0.41). This suggests a potentially different mode of initial interaction, where the positive charge may drive stronger electrostatic attraction to anionic membrane components, while its lower amphipathic character might result in less efficient membrane disruption. This could explain its higher IC_50_ (39.97 µM) compared to PP1 (17.08 µM) against A375 cells, indicating that while both peptides are active, the combination of near-neutral charge with high amphipathicity (as in PP1) appears more optimal for membranolytic action. The biophysical profiling (Table S1) also offers insights into the peptides’ potential mode of action. While the positive ΔG-Interface values and modest hydrophobic moments suggest these peptides are not strong, canonical membrane-disrupting agents, the differential profile of the active compounds is noteworthy. PP1 and PP5, which showed selective bioactivity, possess the lowest ΔG-Interface and negative Boman indices. This combination indicates a relatively higher propensity for lipid interaction and a lower intrinsic protein-binding potential compared to their inactive counterparts. This profile may allow for a selective, although potentially weaker, interaction with the disordered membranes of target cells or facilitate an alternative mechanism.

The selective cytotoxicity of PP1 and PP5 against A375 melanoma cells, in contrast to their limited effects on other cancer lines, points to the critical role of specific membrane characteristics of the target cells. Malignant melanoma cells, including A375, are reported to exhibit a distinct membrane lipidome, often characterized by higher phosphatidylserine externalization, increased sialic acid content on glycoproteins and gangliosides, and altered lipid raft composition compared to other carcinomas [42,43]. The neutral charge and high amphipathicity of PP1 may render it particularly adept at interacting with these specific anionic and structural components on the A375 membrane, while its interaction with the membranes of other tested cell lines (e.g., MIA PaCa-2, A549) may be insufficient to trigger lytic events [44]. PP5, with its positive charge, might rely more heavily on electrostatic recognition of these anionic surfaces. This highlights the importance of expanding the screening to a wider panel of cancer cell lines with well-defined membrane lipidomic profiles to decipher the precise determinants of selectivity and to further refine structure-activity relationships.

Curiously, the high sequence identity (100%) of PP1, PP2, and PP4 with conserved regions of actin proteins from diverse bivalves and gastropods, as revealed by BLAST analysis and phylogenetic trees (Figure S1), provides crucial insight into their origin [45]. This finding strongly suggests that these peptides are cryptic bioactive fragments, also known as cryptides, bioactive peptides encrypted within the sequence of larger, functionally distinct proteins and released via proteolysis [24,25]. This represents an effective evolutionary strategy; an organism can leverage its abundant proteome as a reservoir for generating defensive peptides on demand, without the need for dedicated biosynthetic pathways for each peptide [25]. Therefore, PP1, PP2, and PP4 are more accurately described as multifunctional cryptides. This distinction does not diminish their therapeutic potential but rather highlights a fascinating origin story and aligns our discovery with a growing paradigm in functional peptidomics.

While the current findings are promising, they are constrained by the scope of the biological screening. Furthermore, the safety assessment, although encouraging based on the lack of hemolysis and cytotoxicity in fetal lung fibroblasts (WI-38), remains preliminary. A definitive safety profile will require comprehensive toxicological evaluation using additional relevant non-malignant cell lines from key organs, including hepatic and renal cell models (such as HepG2 or HEK293), and, ultimately, assessment in appropriate in vivo models to fully establish the therapeutic window before clinical development can be considered. The panel of four bacterial strains and five cancer cell lines, while representative, is insufficient to fully delineate the spectrum of activity. Notably, the in silico predictions suggested antifungal potential for PP2 (ClassAMP) and antiviral potential for PP3 and PP5 (ClassAMP). This warrants experimental validation, particularly against clinically relevant fungal pathogens like *Candida albicans* and *Aspergillus fumigatus*. It is noteworthy that many known antifungal peptides from invertebrates exhibit a degree of hydrophilicity to interact with fungal membrane components like glucans and chitin, which could align with the properties of PP2 (GRAVY: −0.39) and PP4 (GRAVY: −0.89) [19,46,47]. A more comprehensive evaluation should also include clinically isolated multidrug-resistant bacteria to assess the potential of these peptides against contemporary resistant strains.

To unequivocally establish the mechanism of action for PP1 and PP5, a series of targeted experiments is required. The initial step would involve membrane permeability assays using dyes such as SYTOX Green or propidium iodide to confirm membrane disruption in real-time [48,49]. Furthermore, to distinguish between various membranolytic models, calcein leakage experiments using large unilamellar vesicles (LUVs) with lipid compositions mimicking cancer versus normal cell membranes would provide critical insights [50,51]. Confocal microscopy with fluorescently labeled peptides would determine their subcellular localization and potential for internalization, and interaction with intracellular targets [52,53]. For the anticancer activity, subsequent assays to assess apoptosis induction via Annexin V binding and caspase activation would clarify whether membrane disruption is the primary cause of cell death or the activation of apoptotic signaling [54,55]. From a translational perspective, PP1 and PP5 provide robust foundational templates for the development of peptide-based therapeutics. To enhance their pharmacological properties, a rational design strategy is necessary. Subsequent efforts could focus on systematic alanine scanning to identify residues critical for activity, followed by strategic substitutions with canonical or non-canonical amino acids to fine-tune hydrophobicity, helicity, and charge [39,56]. It is also important to note that the favorable proteolytic stability and half-life reported are based solely on in silico predictions (HLP server). Experimental validation of these peptides’ stability in biological fluids (such as serum, simulated gastric/intestinal fluid) and their pharmacokinetic profile in vivo are essential future steps to fully assess their therapeutic potential and guide formulation strategies.

In conclusion, this investigation validates *Pisania pusio* as a source of multifunctional peptides with therapeutic potential. PP1 and PP5 have been identified as lead compounds with demonstrable selective cytotoxicity and antimicrobial activity, coupled with a low toxicity profile in vitro. Their novel sequences and initial functional data justify a more extensive investigation into their mechanisms of action and the initiation of a structure-activity relationship (SAR) study to develop optimized analogues with enhanced potency and stability for potential application against resistant infections and specific cancers.

## 4. Materials and Methods

### 4.1. Invertebrate Collection and Sample Preparation

We collected eight individuals of the marine mollusk *Pisania pusio* at Girón Beach, Matanzas, Cuba. All specimens were frozen before separating the shell from the body of the snails. Soft parts (foot and visceral mass) were homogenized in PBS using a blender. The homogenate was centrifuged at 10,000× *g* for 10 min at 4 °C, and the supernatant was ultra-filtrated with a molecular weight cut-off of 10 kDa at 3000 *g* for 10 min at 4 °C. The low-molecular-weight fraction was lyophilized and stored at −20 °C for further LC-MS/MS analysis. The species used in this study is not endangered nor subjected to any regulation and is widely distributed throughout Cuba.

### 4.2. Peptide Sequencing Analysis with NanoLC-ESI-MS-MS

*Pisania pusio* sample was treated with 5 mM DTT for 20 min at RT, followed by incubation with iodoacetamide for 20 min at 37 °C. The samples (15 µL) were analyzed using an Orbitrap Elite Hybrid mass spectrometry system (Thermo Fisher Scientific, Bremen, Germany) online coupled to a U3000 RSLCnano UPLC (Thermo Fisher Scientific, Waltham, MA, USA) as previously described [57]. The raw data analysis was performed with PEAKs 12 software (http://www.bioinfor.com/peaks-software/) [57] (accessed on 15 May 2025), using the DeepNovo Peptidome workflow comprising de novo sequencing (deep-learning-based) combined with UniProtKB reviewed (*Mollusca*) database search (http://www.uniprot.org, accessed on 15 May 2025). For the database search, carbamidomethylated cysteine was considered a fixed modification along with oxidation (M) as a variable modification. In total, eleven peptides, representing nine non-redundant sequences of 8–12 amino acids, were identified by PEAKs 12 using a false discovery rate of 1% and a minimum DeepNovo score of 70% [58]. Peptide sequences and their respective proteins of origin are shown in Supplementary Table S2. Following identification, the novel peptide sequences were subjected to BLAST analysis against the UniProt and NCBI non-redundant protein databases restricted to the *Mollusca* taxon to determine their potential protein precursors and assess if they represented fragments of larger proteins (cryptides) or novel sequences [44]. The BLAST analyses were performed via the Uniprot (https://www.uniprot.org/blast) (accessed on 15 May 2025) and NCBI (https://blast.ncbi.nlm.nih.gov) (accessed on 15 May 2025) web interfaces, both accessed on 15 May 2025, using their corresponding default parameters: (Uniprot: E-value = 10, BLOSUM62 matrix) and (NCBI: E-value threshold = 0.05, BLOSUM62 matrix, gap penalities: existence 11, extension 1). Sequences displaying significant homology were used to construct phylogenetic trees, elucidating their evolutionary relationships within the phylum (Figure S1).

### 4.3. Bioinformatics Analysis for the Prediction of Biological Activities and Candidate Selection

To systematically identify and prioritize candidate peptides with dual potential from the *P. pusio* peptidome, a sequential in silico screening pipeline was employed. This process began with 11 unique peptide sequences identified by nanoLC-ESI-MS/MS (Supplementary Table S1). The selection was based on established computational criteria for bioactive peptides, focusing on predicted multifunctionality, safety, and stability [28,31,33,37,39].

Candidates were required to show a predicted antimicrobial activity (AMP) score of ≥0.6 from at least one of the following servers: ClassAMP (http://www.bicnirrh.res.in/classamp/) (accessed on 15 May 2025) [28], CAMPR3 (http://www.camp3.bicnirrh.res.in/predict) (accessed on 15 May 2025) [29], and AMP Scanner vr.2 (https://www.dveltri.com/ascan/v2/ascan.html) (accessed on 15 May 2025) [30]. Concurrently, a prediction of anticancer activity (ACP) was required, defined as a score ≥ 0.6 in either the AntiCP 2.0 (https://webs.iiitd.edu.in/raghava/anticp2/) (accessed on 15 May 2025) [31] or CancerPPD2 (http://webs.iiitd.edu.in/raghava/cancerppd2/) (accessed on 15 May 2025) [32] servers.

Peptides meeting these bioactivity thresholds were then filtered for key developability parameters. To ensure a favorable preliminary safety profile and focus on membranolytic mechanisms, candidates were required to be predicted as Non-Toxic by ToxinPred (https://webs.iiitd.edu.in/raghava/toxinpred/design.php) (accessed on 15 May 2025) [33] and Non-Cell-Penetrating by CellPPD (https://webs.iiitd.edu.in/raghava/cellppd/algo.php) (accessed on 15 May 2025) [34]. Furthermore, to identify sequences with potential oral stability, a predicted half-life > 1.0 s in a gut-like environment was required, as determined by the HLP server (https://webs.iiitd.edu.in/raghava/hlp/) (accessed on 15 May 2025) [35].

The five peptides (PP1–PP5) were selected as they uniquely satisfied this complete set of filters. This rational and reproducible workflow ensured the selected candidates had a strong in silico profile supporting their experimental validation as multifunctional leads.

To gain deeper insight into the potential mechanism of action, key biophysical parameters related to membrane interaction and protein binding were calculated. The Wimley-White whole-residue interfacial hydrophobicity (ΔG-Interface) and the Boman index (protein-binding potential) were obtained for all peptides using the prediction tools available in the Antimicrobial Peptide Database (APD3; https://aps.unmc.edu/prediction) (accessed on 15 May 2025) [59]. The hydrophobic moment (µH), a quantitative measure of amphipathicity in an α-helical conformation, was calculated using the EMBOSS hmoment tool (https://www.bioinformatics.nl/cgi-bin/emboss/hmoment) (accessed on 15 May 2025) with the Eisenberg hydrophobicity scale and assuming an ideal α-helical structure (100° rotation per residue). The analysis window was set to the full length of each peptide. Helical wheel projections were generated for peptides with sufficient length using the HeliQuest server (https://heliquest.ipmc.cnrs.fr) (accessed on 15 May 2025) [60] under the same helical parameters.

### 4.4. Peptide Synthesis

The peptides were synthesized in solid-phase using the Fmoc/tBu 9-fluorenyl methoxycarbonol methodology and purified by reverse-phase high-performance liquid chromatography (HPLC) to >95% purity using an acetonitrile/H_2_O-TFA gradient (Figure S2). Purity was confirmed by ion spray mass spectrometry (Micromass, Manchester, UK), and molecular mass was confirmed by ion nebulization mass spectrometry (Functional Peptidomics Laboratory, University of Ulm, Ulm, Germany) (Figure S3). All the peptides were synthesized with the C-terminus amidated for greater stability.

### 4.5. Hemolytic Activity Assay

Hemolytic activity was evaluated by measurement of hemoglobin release. Fresh human blood was extracted from a healthy donor at the Clinic University of Ulm, Germany, and stabilized with Heparin. The whole blood sample was washed with PBS by centrifugation at 3000× *g* for 5 min at 4 °C, and the plasma was discarded. The washing procedure was repeated three times until the supernatant was clear, allowing the isolation of hRBC. The remaining pellet was then re-suspended in PBS to obtain a 1% erythrocyte suspension. The PP peptides were prepared at an initial concentration of 800 μM in PBS, added to the first well of a 96-well plate, and diluted serially by a factor of 1/2, resulting in a final volume of 100 µL of sample in every well. PBS and 1% of Triton X-100 (100 µL) were used as negative and positive controls, respectively. A volume of 100 µL of hRBC suspension was transferred to the peptide solutions in a V-bottom 96-well plate, achieving a final peptide concentration range of 100–1 μM, and then incubated for 2 h at 37 °C. The plate was centrifuged, and 100 µL of the supernatant was carefully transferred to a flat-bottom 96-well plate. The absorbance was measured at 540 nm with a microplate reader (Tecan Infinite F200 microplate reader (Tecan Group Ltd., Männedorf, Switzerland). The hemolytic activity was expressed by the hemolysis percentage as follows: hemolysis percentage = (OD (test) − OD (negative control))/(OD (positive control) − OD (negative control)) × 100%.

### 4.6. Microorganism Strains and Growth Conditions

Four bacterial species were used to evaluate the antimicrobial activity of the AMPs: *Pseudomonas aeruginosa* ATCC 15692, *Klebsiella pneumoniae* ATCC 10031, *Staphylococcus aureus* ATCC 29737, and *Escherichia coli* ATCC 10536. Strains were maintained on Mueller–Hinton Agar (MHA) plates and subcultured no more than twice before each experiment. For antimicrobial testing, a single colony of the bacterial strains was grown in 5 mL Müller–Hinton broth (Carl Roth GmbH (Karlsruhe, Germany)) liquid cultures for 16 h at 180 rpm at 37 °C.

### 4.7. Determination of Antimicrobial Activity

The minimum inhibitory concentration (MIC) of the peptides was determined against a panel of clinically relevant bacterial strains in Mueller-Hinton broth using the standard broth microdilution method according to the Clinical and Laboratory Standards Institute (CLSI) guidelines (M27-A3 broth microdilution assay). Bacterial strains were cultured overnight in Müller–Hinton broth (MHB) at 37 °C with agitation. The bacterial inoculum was adjusted to a concentration of approximately 5 × 10^6^ colony-forming units (CFU)/mL in fresh MHB. A two-fold serial dilution of each peptide was prepared in sterile, low-binding 96-well polystyrene microplates starting in 200 μM with a volume of 100 µL. Subsequently, 100 µL of the bacterial inoculum was added to each well, resulting in a final volume of 200 µL and a final peptide concentration range of 100 to 1.5625 μM. The final bacterial density in each well was 5 × 10^5^ CFU/mL. Control wells were included in each assay: negative control (bacteria + MHB, without peptide), sterility control (MHB only), and positive control using Gentamicin. After 24 h of incubation at 37 °C, the OD at 600 nm was measured using a Tecan microplate reader M infinite 200 (Tecan Group Ltd., Männedorf, Switzerland), and the MIC was determined visually as the lowest peptide concentration that completely inhibited visible bacterial growth. All assays were performed in triplicate on three independent occasions (*n* = 3).

### 4.8. Human Cells and Culture Conditions

The human cancer cell lines, A549 (epithelial cell lung carcinoma, ATCC number CRM-CCL-185), A375 (malignant melanoma, ATCC number CRL-1619), MIA PaCa-2 (pancreatic carcinoma, ATCC number CRM-CRL-1420), MCF-7 (luminal A breast adenocarcinoma, non-invasive, ATCC number HTB-22), and MDA-MB-231 (triple negative basal breast adenocarcinoma, invasive, ATCC number HTB-26) were used for cytotoxicity experiments to evaluate the anticancer activity of the peptides. On the other hand, the human diploid cell line WI-38 (fetal lung epithelial fibroblasts, ATCC number CCL-75) was used to evaluate toxicity in non-malignant human cells. The cells were obtained from the ATCC (American Type Culture Collection, Manassas, VA, USA). All cell lines were cultured in DMEM (Dulbecco’s Modified Eagle’s Medium, Gibco, Paisley, UK) supplemented with 10% heat-activated fetal bovine serum (FBS, Gibco, Paisley, UK), 2 mM L-glutamine, 1 mM sodium pyruvate, 18 mM HEPES, 26 mM NaHCO_3_, and 50 µg/mL penicillin/streptomycin solution (Gibco, Paisley, UK), and maintained at 37 °C in a 5% CO_2_ atmosphere.

### 4.9. Cytotoxicity Assays

The cytotoxic activity of the peptides was assessed by determining cell viability with the resazurin reduction assay (Merck, Darmstadt, Germany). In brief, 5 × 10^4^ cells per well were distributed in a 96-well plate and incubated overnight at 37 °C to allow adherence. After removing the medium, cells were treated with the peptides dissolved in serum-free medium at concentrations ranging from 1.5625 to 100 μM for cancer cells and 1.5625–800 μM for WI-38 cells, and then incubated for 24 h at 37 °C in a 5% CO_2_ atmosphere. Subsequently, 20 µL of resazurin solution at 0,15 mg/mL was added to each well, for further incubation protected from the light during 4 h at 37 °C. Finally, fluorescence of the transformed resorufin was measured at an excitation wavelength of 535 nm and an emission wavelength of 595 nm using a TECAN infinite M200 microplate reader (Tecan Group Ltd., Männedorf, Switzerland). Cells treated with medium were used as a negative control and set as 100% viability, whereas cells treated with Triton X-100 at 1% were used as a positive control. The experiments were performed in triplicate, and the results show the average of three independent experiments. IC_50_ values (peptide concentration that reduces cell viability by 50%) were determined by fitting the dose-response data to a four-parameter logistic (4PL) nonlinear regression model using GraphPad Prism (version 8.0) (GraphPad Software, La Jolla, CA, USA).

### 4.10. Statistical Analysis

All data represent the mean ± standard deviation (SD) of three independent experiments. Differences between the two groups were analyzed using a two-tailed Student’s *t*-test, and comparisons between multiple groups were performed using analysis of variance (ANOVA) and Dunnett’s test. A probability value (*p*) < 0.05 was taken as the threshold for significant differences. Statistical analyses and graphs were performed using GraphPad Prism (version 8.0) (GraphPad Software, La Jolla, CA, USA).

### 4.11. Calculation of in Vitro Selectivity Indices

A quantitative assessment of selectivity was performed to define the therapeutic potential of the peptides. Since neither hemolysis nor cytotoxicity in WI-38 fibroblasts reached 50% at the highest concentration tested (800 µM, see Figure 2 and Figure 3A), the standard half-maximal toxicity values (HC_50_ and IC_50_, respectively) are >800 µM for all peptides. Therefore, exact selectivity indices based on HC_50_ or IC_50_ ratios could not be determined. Instead, conservative minimum selectivity indices were calculated using 800 µM as a robust, lower-limit proxy for significant toxicity, providing a stringent estimate of the in vitro therapeutic window.

For antimicrobial activity, the Minimum Hemolytic Selectivity Index (HSI_min_) was calculated as:

$$HSI_{min}=\frac{800\;\mu M}{MIC}$$

where MIC is the minimum inhibitory concentration against a given bacterial strain. This index represents the minimum fold-difference between the concentration causing significant hemolysis and the antimicrobial effective concentration.

For the anticancer lead peptides PP1 and PP5, the Minimum Cytotoxicity Selectivity Index (CSI_min_) was calculated as:

$$CSI_{min}=\frac{800\;\mu M}{{\mathrm{IC}}_{50}(A375)}$$

where IC_50_(A375) is the half-maximal inhibitory concentration against A375 melanoma cells. The CSI_min_ represents the minimum window between toxicity to normal fibroblasts and efficacy against cancer cells.

## 5. Conclusions

This study establishes the marine gastropod *Pisania pusio* as a novel source of bioactive peptides. The integrated discovery pipeline identified PP1 and PP5 as lead compounds, demonstrating selective anticancer activity against melanoma cells and moderate antimicrobial effects, while showing low toxicity in initial in vitro assays. These findings highlight the potential of these peptides as promising templates for further optimization and preclinical investigation as new therapeutic agents against cancer and bacterial infections.

## Figures and Tables

**Table 1:** Physicochemical properties of *P. pusio* peptides calculated with ExPASy ProtParam.

Peptide	Sequence	Mol WT	Charge (pH 7)	Hydrophobicity (GRAVY)	Amphipathicity	PI
PP1	IKIIAPPE	880.21	0	0.59	0.62	6.35
PP2	LRVAPEEHPV	1146.45	−0.90	−0.39	0.64	5.41
PP3	DVGWALKPAK	1084.42	1.00	−0.26	0.73	8.94
PP4	IVTNWDDMEK	1250.53	−2.00	−0.89	0.49	4.03
PP5	KLLSASPPL	925.26	1.00	0.50	0.41	9.11

**Table 2:** In silico prediction of antimicrobial and anticancer activity for *P. pusio*-derived peptides.

		ClassAMP		CAMPR3		AMP Scanner vr.2		AntiCP 2.0		Cancer PPD2	Cell PPD	ToxinPred	HLP	
Peptide	Pred	Score	Pred	Score	Pred	Score	Pred	Score	Pred	Score	Pred	Pred	Pred.HL-sec	Pred. Stability
PP1	AB	0.870	AB	**0.682**	No AMP	0.043	ACP	**1.0**	ACP	**0.74**	Non-CPP	Non-Toxic	1.192	High
PP2	AF	0.775	AB	0.464	No AMP	0.014	ACP	**0.98**	ACP	0.48	Non-CPP	Non-Toxic	1.63	High
PP3	AV	**0.978**	AB	**0.9**	**AMP**	**0.908**	ACP	**0.95**	ACP	**0.73**	Non-CPP	Non-Toxic	**2.582**	High
PP4	AB	**0.919**	AB	0.55	No AMP	0.194	ACP	0.65	ACP	0.54	Non-CPP	Non-Toxic	1.02	High
PP5	AV	**0.895**	AB	**0.714**	**AMP**	**0.548**	ACP	0.5	ACP	**0.60**	Non-CPP	Non-Toxic	**2.479**	High

For each server (column), the table exhibits the corresponding prediction (left) and score (right) assigned to the peptides. The higher values in each classification were highlighted in bold. AB: antibacterial, AF: antifungal, AV: antiviral, ACP: anticancer peptide, Non-CPP: non-cell penetrating peptide, Pred. HL-sec: Half Life in seconds.

**Table 3:** MIC in μM for each peptide against different species of bacteria at 24 h of treatment.

Bacterial Strain	PP1	PP2	PP3	PP4	PP5
*Pseudomonas aeruginosa* ATCC 15692	100	100	100	100	100
*Klebsiella pneumoniae* ATCC 10031	100	>100	>100	>100	100
*Escherichia coli* ATCC 10536	100	100	100	100	100
*Staphylococcus aureus* ATCC 29737	25	>100	>100	>100	>100

**Table 4:** IC_50_ values in μM for each peptide in different cell lines at 24 h of treatment.

Cell Lines	PP1	PP2	PP3	PP4	PP5
WI-38	>800	>800	>800	>800	>800
A375	17.08 ± 1.36	>100	>100	>100	39.97 ± 1.29
MDAMB-231	76.25 ± 1.50	>100	>100	>100	>100
MCF-7	98.53 ± 1.34	>100	>100	>100	>100
A549	>100	>100	>100	>100	>100
MIA PaCa-2	>100	>100	>100	>100	>100

IC_50_ (peptide concentration that reduces cell viability by 50%) in μM of each peptide at 24 h in the WI-38 and cancer cell lines, determined by fitting the dose-response data to a four-parameter logistic nonlinear regression model. IC_50_ values that exceeded the concentrations evaluated in the assays are represented as >800 or >100 μM for WI-38 and cancer cells, respectively.

**Table 5:** Minimum Hemolytic Selectivity Index for the antimicrobial activity of *P. pusio* peptides.

Peptide	*P. aeruginosa*	*K. pneumoniae*	*E. coli*	*S. aureus*
PP1	>8	>8	>8	>32
PP2	>8	n.a	>8	n.a
PP3	>8	n.a	>8	n.a
PP4	>8	n.a	>8	n.a
PP5	>8	>8	>8	n.a

n.a: not applicable (no antimicrobial activity against this strain at tested concentrations).

**Table 6:** Minimum Cytotoxic Selectivity Index of *P. pusio* peptides.

Peptide	IC_50_ (A375)	Cytotoxicity SI-min (vs. WI-38)
PP1	17.08	>46.8
PP5	39.97	>20.0

## Data Availability

Data is contained within the article or Supplementary Material.

## Supplementary Materials

The following are available online at https://www.mdpi.com/article/10.3390/md24010032/s1. Figure S1: Phylogenetic trees of PP peptides constructed from an analysis of homologous sequences identified by BLAST against the NCBI database. Figure S2: Purity analysis of synthesized peptides by analytical HPLC. Figure S3: Mass spectrometry analysis of synthesized peptides. Figure S4: Helical wheel projections of *Pisania pusio*-derived peptides PP2, PP3, and PP4. Table S1: Biophysical parameters of *P. pusio* peptides. Table S2: Peptide sequences and their respective proteins of origin.
