# Supplementary material for: Integrating Computational and Experimental Approaches for the Discovery of Multifunctional Peptides from the Marine Gastropod Pisania pusio with Antimicrobial and Anticancer Properties
Source: Mar Drugs. 2026 Jan 8;24(1):32. doi: 10.3390/md24010032 (PMC12843047; doi:10.3390/md24010032)
Supplement: Supplementary file 1 [file marinedrugs-24-00032-s001.zip › marinedrugs-4029629-supplementary.pdf]

marine drugs

MDPI

Article

Integrating Computational and Experimental Approaches for the Discovery of Multifunctional Peptides from the Marine Gastropod *Pisania pusio* with Antimicrobial and Anticancer Properties

Ernesto M. Martell-Huguet [1,2], Thalia Moran-Avila [2], José E. Villuendas [2], Armando Rodriguez [3,4], Ann-Kathrin Kissmann [1], Ludger Ständker [4], Sebastian Wiese [3], Anselmo J. Otero-Gonzalez [2,*] and Frank Rosenau [1,*]

1 Institute of Pharmaceutical Biotechnology, Ulm University, 89081 Ulm, Germany; nestmartell@gmail.com (E.M.M.-H.); ann-kathrin.kissmann@uni-ulm.de (A.-K.K.)
2 Center for Protein Studies, Faculty of Biology, University of Havana, 25 and I, La Habana 10400, Cuba; moravilat@gmail.com (T.M.-A.); joseenriquevilluendasquesada@gmail.com (J.E.V.)
3 ULMTec Core Facility of Mass Spectrometry and Proteomics, Faculty of Medicine, Ulm University, Albert-Einstein-Allee 11, 89081 Ulm, Germany; armando.rodriguez-alfonso@uni-ulm.de (A.R.); sebastian.wiese@uni-ulm.de (S.W.)
4 ULMTec Core Facility for Functional Peptidomics, Ulm Peptide Pharmaceuticals (U-PEP), Faculty of Medicine, Ulm University, 89081 Ulm, Germany; ludger.staendker@uni-ulm.de
* Correspondence: aotero@fbio.uh.cu (A.J.O.-G.); frank.rosenau@uni-ulm.de (F.R.)

1. Supplementary Materials

Additional Figures and Tables

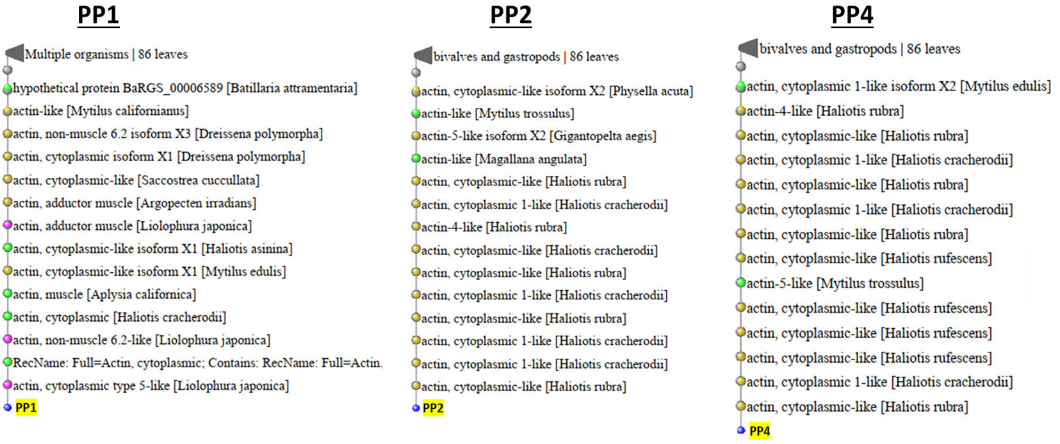

Figure S1. Phylogenetic trees showing the homology relationship of *Pisania pusio* peptides PP1, PP2 and PP4 with actin sequences from mollusks. The trees were constructed from an analysis of homologous sequences identified by BLAST against the NCBI database, restricted to the *Mollusca* taxon. The observed clustering confirms the high conservation of these peptide sequences within structural proteins of bivalves and gastropods. Peptides PP3 and PP5 are not shown as their short sequences produced ambiguous phylogenetic trees with homologies distributed across multiple unrelated protein families, lacking the clear evolutionary relationship observed for the actin-derived peptides.

Academic Editor(s): Name

Received: date
Revised: date
Accepted: date
Published: date

Copyright: © 2026 by the authors. Submitted for possible open access publication under the terms and conditions of the Creative Commons Attribution (CC BY) license.

PP1

IKIIAPPE

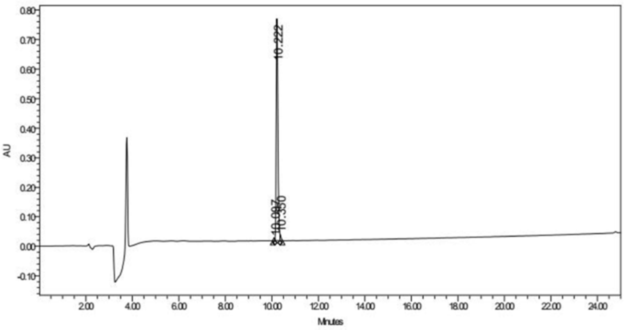

	RT	Area	% Area	Height
1	10.097	34103	0.87	9044
2	10.222	3813744	97.44	747251
3	10.350	66279	1.69	21073

PP2

LRVAPEEHPVV

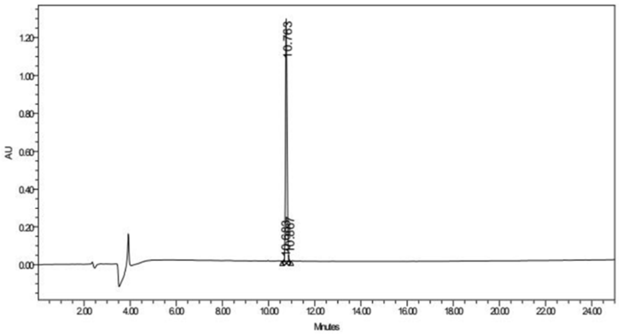

	RT	Area	% Area	Height
1	10.683	19390	0.31	10471
2	10.763	6202452	99.14	1274635
3	10.867	34644	0.55	29446

PP3

DVGWALKPAK

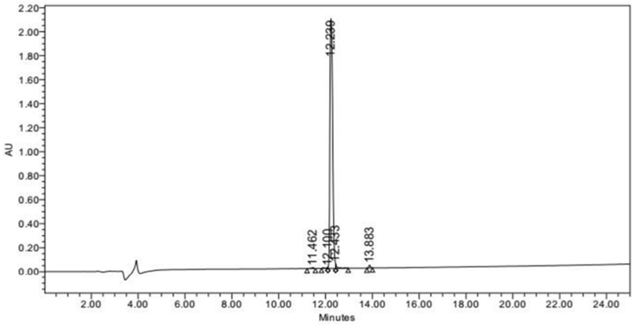

	RT	Area	% Area	Height
1	11.462	61845	0.35	7389
2	12.100	54875	0.31	8236
3	12.239	17101930	97.81	2098524
4	12.433	147677	0.84	38985
5	13.883	118008	0.67	23550

PP4

IVTNWDDMEK

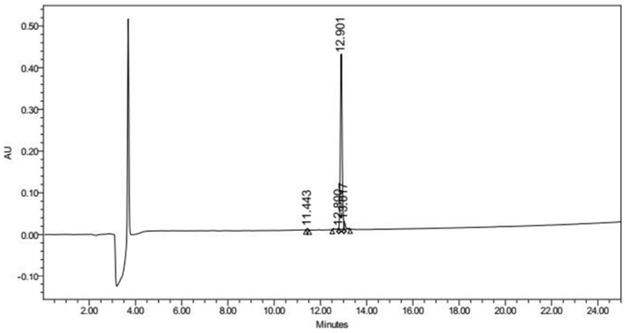

	RT	Area	% Area	Height
1	11.443	14798	0.65	3230
2	12.800	16777	0.73	3188
3	12.901	2179903	95.07	414781
4	13.017	81381	3.55	18608

PP5

KLLSASPPL

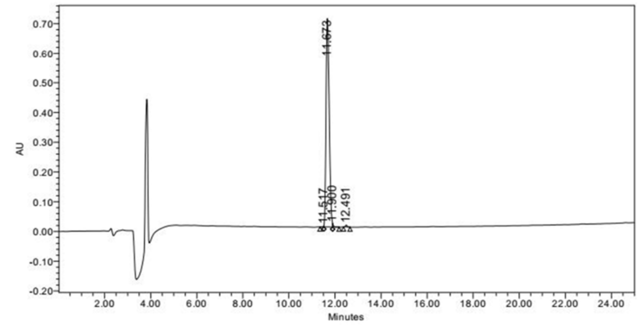

	RT	Area	% Area	Height
1	11.517	5659	0.08	4014
2	11.673	6641751	98.94	704578
3	11.900	28575	0.43	11702
4	12.491	36884	0.55	6027

Figure S2. Purity analysis of synthesized peptides by analytical HPLC. Chromatograms are shown for peptides PP1, PP2, PP3, PP4, and PP5. The retention time (RT, min) and the relative peak area (% Area) for the main product are indicated within each panel. The high purity of each peptide (>95%) is demonstrated by the dominance of the major peak and the minimal area contribution from minor impurities.

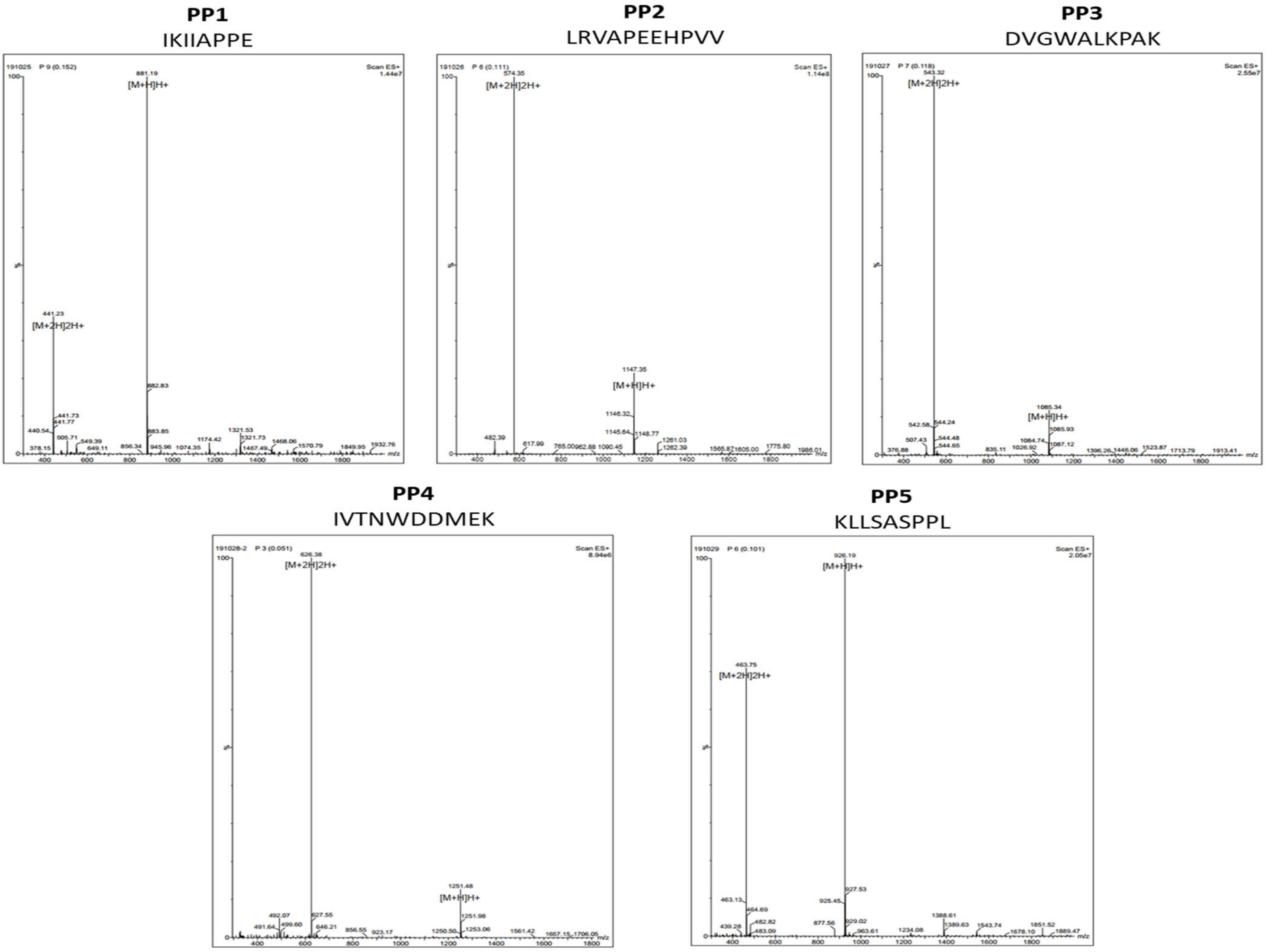

Figure S3. Mass spectrometry analysis of synthesized peptides. ESI mass spectra confirming the molecular weight of the synthesized and purified PP peptides. The observed monoisotopic masses [M+H]⁺ correspond to the theoretical masses of the target peptides with C-terminal amidation.

Table S1. Biophysical parameters of *P. pusio* peptides

Peptide	Sequence	ΔG-Interface (kcal/mol)	Boman Index (kcal/mol)	Hydrophobic Moment (μH)
PP1	IKIIAPPE	3.15	-0.52	0.326
PP2	LRVAPEEHPV	5.67	1.83	0.293
PP3	DVGWALKPAK	1.67	0.39	0.330
PP4	IVTNWDDMEK	3.71	2.53	0.483
PP5	KLLSASPPL	0.64	-0.46	0.310

[1] Calculated biophysical parameters of *Pisania pusio*-derived peptides. ΔG-Interface (Wimley-White interfacial hydrophobicity) and the Boman index (protein-binding potential) were predicted using the APD3 database. The hydrophobic moment (μH) was calculated for an ideal α-helix (100° rotation/residue) using the EMBOSS hmoment tool with the Eisenberg scale.

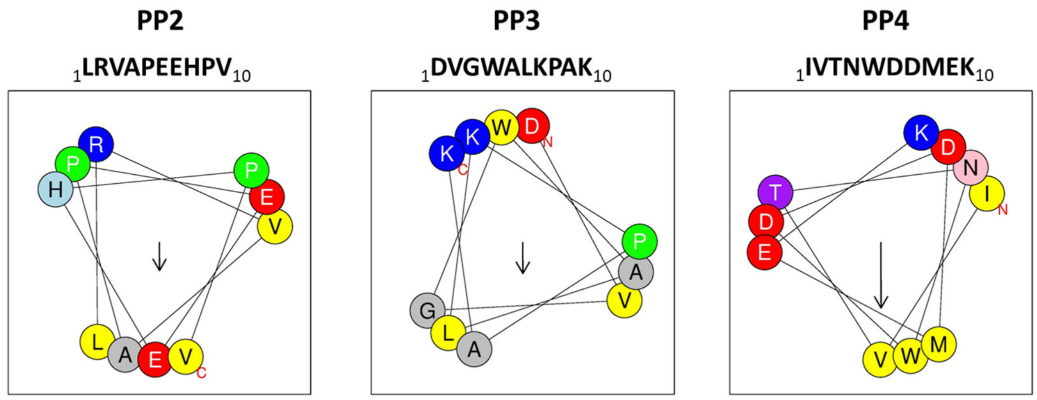

Figure S4. Helical wheel projections of *Pisania pusio*-derived peptides PP2, PP3, and PP4. Projections were generated using the HeliQuest server, assuming an ideal α-helical conformation (100° rotation per residue). The color code of residues as displayed by the server is as follows: hydrophobic (yellow), polar (cyan), positively charged (blue), negatively charged (red), glycine/proline/special (green), and other/neutral (gray). These visualizations highlight the spatial segregation of residues, contributing to the peptides' amphipathic character.

Table S2. Peptide sequences and their respective proteins of origin

Peptide	Accession	Found By
IVTNWDDMEK	Q964E1|ACTC_BIOOB:Q964E2|ACTC_BIOPF:Q964E3|ACTC_BIOAL:Q26065|ACT_PLAMG:Q964D9|ACTC_PLATR:P17304|ACTM_APLCA:Q964E0|ACTC_BIOTE:O17320|ACT_CRAGI:P92179|ACTC_BIOGL	DB Search
GI-VTNWDDMEK	Q964E1|ACTC_BIOOB:Q964E2|ACTC_BIOPF:Q964E3|ACTC_BIOAL:Q26065|ACT_PLAMG:Q964D9|ACTC_PLATR:P17304|ACTM_APLCA:Q964E0|ACTC_BIOTE:O17320|ACT_CRAGI:P92179|ACTC_BIOGL	DB Search
LRVAPEEHPV	Q964E1|ACTC_BIOOB:Q964E2|ACTC_BIOPF:Q964E3|ACTC_BIOAL:Q26065|ACT_PLAMG:Q92193|ACT_CRAVI:Q964D9|ACTC_PLATR:Q964E0|ACTC_BIOTE:O17320|ACT_CRAGI:P92179|ACTC_BIOGL	DB Search
IVTNWDDM(+15.99)EK	Q964E1|ACTC_BIOOB:Q964E2|ACTC_BIOPF:Q964E3|ACTC_BIOAL:Q26065|ACT_PLAMG:Q964D9|ACTC_PLATR:P17304|ACTM_APLCA:Q964E0|ACTC_BIOTE:O17320|ACT_CRAGI:P92179|ACTC_BIOGL	DB Search
GI-VTNWDDM(+15.99)EK	Q964E1|ACTC_BIOOB:Q964E2|ACTC_BIOPF:Q964E3|ACTC_BIOAL:Q26065|ACT_PLAMG:Q964D9|ACTC_PLATR:P17304|ACTM_APLCA:Q964E0|ACTC_BIOTE:O17320|ACT_CRAGI:P92179|ACTC_BIOGL	DB Search
LKEAPGPLN	P13543|MLR_AR-GIR:P05963|MLR_CHLNI:P04113|MLRA_MIZYE:P04112|MLRB_MIZYE:P05944|MLRC_MIZYE:P02613|MLR_PATSP	DB Search
MLKEAPGPLNF	P13543|MLR_AR-GIR:P05963|MLR_CHLNI:P04113|MLRA_MIZYE:P04112|MLRB_MIZYE:P05944|MLRC_MIZYE:P02613|MLR_PATSP	DB Search
DVGWALKPAK		DeepNovo
LVVDNGSGM	Q964E1|ACTC_BIOOB:Q964E2|ACTC_BIOPF:Q964E3|ACTC_BIOAL:Q26065|ACT_PLAMG:Q964D9|ACTC_PLATR:P17304|ACTM_APLCA:Q964E0|ACTC_BIOTE:O17320|ACT_CRAGI:P92179|ACTC_BIOGL	DB Search
KLLSASPPL		DeepNovo
IKIIAPPE	Q964E1|ACTC_BIOOB:Q964E2|ACTC_BIOPF:Q964E3|ACTC_BIOAL:Q26065|ACT_PLAMG:Q964D9|ACTC_PLATR:P17304|ACTM_APLCA:Q964E0|ACTC_BIOTE:P92179|ACTC_BIOGL	DB Search

[2] **Unique peptide sequences identified from *Pisania pusio* peptidomic analysis and their potential protein precursors.** For each peptide, the corresponding UniProtKB accession numbers (where available) of homologous proteins identified by BLAST search against the *Mollusca* taxon are listed, separated by a vertical bar.

https://doi.org/10.3390/xxxxx
